# Base-excision repair increases DNA double-strand break clustering within heavy-ion tracks and modulates repair at δ-electron-induced breaks

**DOI:** 10.1038/s41598-025-32823-z

**Published:** 2026-01-09

**Authors:** Laura Schwan, Nicole B. Averbeck, Marco Durante, Burkhard Jakob

**Affiliations:** 1 Department of Biophysics, GSI Helmholtzzentrum für Schwerionenforschung GmbH, Planckstr. 1, 64291 Darmstadt, Germany; 2 Department of Biology, Technische Universität Darmstadt, Darmstadt, Germany; 3 Department of Condensed Matter Physics, Technische Universität Darmstadt, Darmstadt, Germany

**Keywords:** Clustered DNA-damage, DNA repair, DNA-end resection, Heavy ions, δ-electrons, Space radiation, Base excision repair, Double-strand DNA breaks, Homologous recombination

## Abstract

Space radiation poses a threat to human health during space missions. Its biological effect largely depends on heavy ions. These induce highly clustered DNA damage along their tracks, which is difficult to repair. If this damage is not repaired correctly, or not at all, mutations and possibly cancer can occur in the long term. δ-electrons induced by fast heavy ions lead to further DNA damage outside ion tracks, resembling that of sparsely-ionising radiation. Using inhibitors of the crucial base-excision repair factors OGG1 and APE1, we show that the repair of DNA base-lesions within heavy-ion tracks causes DNA double-strand breaks (DSBs), which increases difficult-to-repair in-track DSB clustering. We further found that DSBs induced by δ-electrons of fast heavy ions are more often decorated by the resection factor RPA, which suggests that they are more often repaired in a resection-dependent manner than X-ray-induced DSBs, despite their resemblance. Using γH2AX assays to assess DSB repair kinetics, we found that δ-electron-induced DSBs are repaired faster than those induced by X-rays in G1-phase cells, despite the fact that δ-electron-induced DSBs are frequently resected, which typically entails slower repair processes. These findings on δ-electron-induced DSBs imply that the quantity of clustered DSBs in irradiated cells affects the overall response of cells to DNA-damage. Based on our results on base-excision repair and the processing of δ-electron-induced DSBs, we conclude that the interplay between DNA-damage repair processes is a pivotal factor in the course of DNA repair and, consequently, genomic integrity.

**Supplementary Information:**

The online version contains supplementary material available at 10.1038/s41598-025-32823-z.

## Introduction

The complexity of DNA damage is a crucial factor for the fidelity of DNA repair. It is defined by clustering of DNA damage; if there are two or more DNA lesions within one helical turn of the DNA molecule, the damage is considered complex^1^. With increasing clustering, the repairability of DNA damage decreases, and the choice of pathways to eliminate the severe damage of DNA-double-strand breaks (DSBs) changes. Namely, pathways that require DNA-break end processing by resection are more frequently used^2–4^. Canonical Non-homologous end joining (NHEJ), which operates in G1- and G2-phase cells, is the most frequently used and overall fastest pathway to repair DSBs^5^. Another much slower main pathway is resection-dependent homologous recombination (HR), which is active in late S- and G2-phase cells when the required repair template, the sister chromatid, is available^5,6^. Other additional pathways can deal with resected ends like single-strand annealing (SSA) in G2-phase cells and alternative end-joining (aEJ) in G1-, G2-, and M-phase cells^7,8^. All pathways that repair resected DSBs except for HR are intrinsically error-prone. Therefore, the repairability of DSBs and the choice of their repair pathway are crucial in maintaining genomic integrity.

Clustered DSBs along an ion’s track are a hallmark of high-LET (Linear Energy Transfer) radiation due to its densely ionising properties^9,10^. This type of radiation is part of space radiation and mainly derives from galactic cosmic rays (GCR) in the form of HZE-particles (*h*igh atomic number (*Z*) and *e*nergy particles), i.e. fast heavy ions. High-LET radiation is an important factor for the cancer risk of space travellers^11^. Mutations caused by non-repaired or mis-repaired radiation-induced DNA damage are considered a source of radiation-related cancer^12^. Therefore, characterising this damage and how it is processed can provide mechanistic insight into radiation-induced carcinogenesis. Simulation-based estimates of DSB yields often do not agree well with experimental DSB assessments, which mostly are derived from DSB surrogate markers like γH2AX or 53BP1^13^. Discrepancies between experimental observations and simulations of DSB numbers might occur due to radiation-induced foci (RIFs) that cannot resolve individual DSBs by common microscopic techniques^14^. Alternatively, the discrepancies are owed to a non-optimal detection time-point when DSBs are repaired already^15^. On the other hand, the biological processing of radiation-induced non-DSB DNA damage that leads to DSBs is not incorporated into models and therefore may represent a cause of underestimation.

Besides DSBs, high-LET radiation causes further types of DNA lesions like DNA single-strand breaks (SSBs) and different DNA-base lesions^10,16^. Depending on their structure, the latter become repaired by nucleotide-excision repair (NER) or base-excision repair (BER)^17^; BER is a very efficient and well conserved repair pathway to remove SSBs and base lesions that arise from oxidation, deamination, alkylation or base removal^18^. This pathway caught our interest, since earlier work of others and our group has revealed that BER of DNA-base damage adds to the number of DSBs within X- or γ-ray irradiated mammalian cells^19,20^ and hence, represents a biological process that causes additional DSBs. If BER operates either on opposite strands simultaneously or opposite a SSB, DSBs can occur because BER processes involve SSBs^18^. As DNA damage within ion tracks is highly clustered, BER processes can be expected to cause additional DSBs and hence, may further add to DSB clustering. Based upon this, we studied whether and in how far BER contributes to the DSB-damage load within high-LET ion tracks.

Besides the influence of DNA-damage clustering on damage processing within the track, we also studied its influence on damage processing outside the ion trajectories. The main DNA damage of heavy ions is caused in the vicinity of their trajectory by direct ionisation or excitation of DNA molecules, so-called direct action. Yet, it is assumed that DSBs caused by their δ-electrons outside the trajectory are also relevant for survival^21,22^. Considering the energy and fluence of the iron (Fe) ions used, the resulting energy spectra of δ-electrons outside the trajectory are quite comparable to sparsely ionising X-rays^23,24^. Therefore, δ-electron-induced DNA lesions are caused by similar action, i.e. indirect action via radicals (mainly from water molecules)^22^, and are of similar quality. The latter was shown in earlier studies, which revealed that δ-electron-induced DSBs are comparable to photon-induced DSBs^20,25^. However, as the DNA-damage clustering within ion tracks is substantial, the complexity of the in-track damage may dictate the DSB-repair processes of the entire cell, thus including δ-electron-induced DSBs. This assumption is supported by observations of synergistic effects in cells that were exposed simultaneously to X-rays and α-particles^26,27^. Based upon this, we studied the repair response at δ-electron-induced DSBs of cells hit by heavy ions in comparison to X-ray-induced DSBs.

Our goal was to elucidate DSB clustering and processing in more detail in cells that were irradiated with heavy ions, which are a biologically relevant fraction of GCR. The revealed data help to clarify the processes that cause radiation-related health hazards like cancer in space travellers. In pursuit of our goal, we aimed to reveal BER’s contribution to DSB clustering in heavy-ion tracks. We found that BER processes lead to an increase in DSB frequency, both within ion tracks and in nuclear regions distant from the track core. In addition, we wanted to assess whether the high incidence of DSB clustering within these tracks influences the repair processes of off-track δ-electron-induced DSBs within the same cell. This is because the increased clustering of DNA damage within ion tracks leads to DSBs being resected more frequently, regardless of the availability of error-free HR, thereby increasing the likelihood of error-prone repair and thus of mutations. We found that the processing of δ-electron-induced DSBs shifts DSB repair to resection-dependent pathways in the presence of complex in-track damage, compared to X-ray-induced DSB repair. Although resection-dependent DSB repair is considered slow, we observed that δ-electron-induced DSBs distant from the track core in G1-phase cells are repaired faster than DSBs caused by X-rays. Our data indicate that not only the quality of each single lesion determines its repair processes but also the entire DNA-damage load and quality within a cell.

## Results

### BER processes increase the DSB load of Fe-ion induced DNA damage

**Fig. 1:**
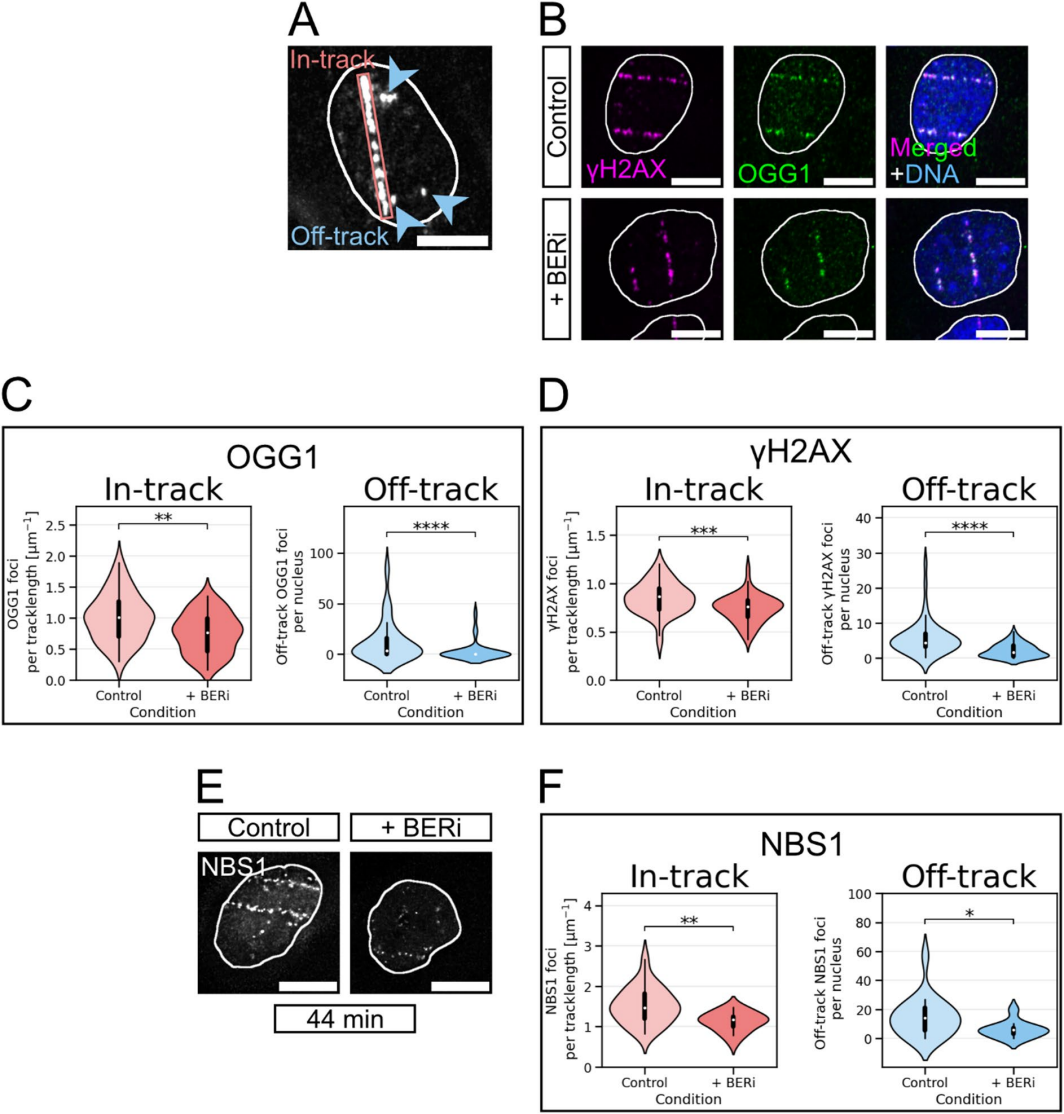
BERi causes less OGG1 and DSB RIF in- and off-track after Fe-ion irradiation. Human fibroblasts (**A – D**) or osteosarcoma cells (E, F) were treated with (+ BERi) or without (control) BER inhibitors and irradiated with Fe ions (350 MeV/n, 3 × 10^6^ p./cm^2^). 15 min after irradiation the cells were fixed for IF microscopy (**A – D**), or live cell imaging was performed just before and during 44 min after irradiation (**E**,** F**). Shown scale bars indicate 10 μm. A Definition of in-track and off-track signals: To discriminate between in- and off-track RIF, DSB markers γH2AX, 53BP1, or NBS1-2GFP were used. In-track: along the trajectory (box), off-track: at least 1 μm distance to the track’s centre (arrows). (**B**) Representative images of fibroblasts IF stained for OGG1 (green) and γH2AX (magenta); DNA was counterstained with DAPI (blue). Nuclei are indicated by white borders. (**C – D**) Distribution of OGG1 (**C**) or γH2AX foci (**D**) per track length (in-track) and per nucleus excluding the track area (off-track). (**E**) Representative images of living NBS1-2GFP expressing osteosarcoma cells 44 min after irradiation. (**F**) Distribution of NBS1-GFP foci per track length (in-track) and per nucleus excluding the track area (off-track). Statistical analyses between the control and inhibitor groups were performed by the non-parametric Mann-Whitney U test: (**C**): in-track OGG1: *p* = 0.0013 and off-track OGG1: *p* = 5.9 × 10^−5^. (**D**): in-track γH2AX: *p* = 0.00046 and off-track γH2AX: *p* = 1.9 × 10^−7^. With *n* = 25 cells per condition per experiment, *N* = 2 independent experiments. E: in-track NBS1-GFP: *p* = 0.0032 and off-track NBS1-GFP: *p* = 0.040. In-track, 10 living control- and 9 BERi-treated cells were analysed. Off-track, 19 control- and 11 BERi-treated cells were analysed. *N* = 1 experiment.

In order to test whether BER adds to the number of DSBs and thus DSB clustering within tracks of high-LET particles, we irradiated human normal fibroblasts (AG1522D) in the presence or absence of BER inhibition (BERi) with iron (Fe) ions, a biologically relevant high-LET component of space radiation. The cells were irradiated nearly horizontally, which enables visualising radiation-induced DNA damage along ion trajectories (in-track) and outside the trajectories (off-track) induced by δ-electrons (Fig. 1A). RIFs of S139 phosphorylated histone H2AX (γH2AX) and 8-OxoG glycosylase 1 (OGG1) served as DSB marker or BER marker, respectively and were visualised by immunofluorescence (IF) staining (Fig. 1B).

In accordance with earlier data^28^, OGG1 foci can be found at Fe-ion induced DNA damage. Within 15 min after irradiation, which is the earliest time point for fixation after the samples became accessible, OGG1 RIF are observable in-track and off-track. This suggests the induction and subsequent early processing of base lesions in the ion trajectories and at DNA damage of δ-electrons.

To inhibit BER, we chose a combination of the OGG1 inhibitor TH5487 and the apurinic/apyrimidinic endonuclease 1 (APE1) inhibitor methoxyamine, since both APE1 and to a weaker extend OGG1 induce SSBs within BER^29^. The inhibitor cocktail at the given concentration clearly reduced OGG1 foci of KBrO_3_-induced DNA-base damage (Supplementary Figure S1), which provides evidence for a successful inhibition of BER. KBrO_3_ is a powerful oxidant and used to induce oxidative DNA damage, e.g. the OGG1 substrate 7,8-dihydro-8-oxoguanine (8-oxoG)^30,31^. The chosen BER-inhibitor conditions caused the amount of OGG1 foci per nucleus to decrease by 83% indicating that the cocktail prevented OGG1 binding. Under these conditions we further saw about 86% fewer γH2AX foci per nucleus, which implies a substantial contribution of the BER-dependent processing of oxidised base lesions in DSB formation (Table 1).

**Table 1:** OGG1 and γH2AX foci directly after KBrO_3_ treatment in human fibroblasts. *N* = 2 independent experiments, *n* = 25–30 cells per condition.

KBrO_3_-induced foci/nucleus	Control	+ BERi
OGG1	52.4 ± 4.5	9.1 ± 0.5
γH2AX	43.0 ± 1.2	6.1 ± 0.5

In the case of Fe-ion induced OGG1 foci, the numbers of both in- and off-track foci were also clearly reduced in the presence of BER inhibitors (Fig. 1C; a summary of the averaged data is shown in Table 2). Off-track, OGG1 foci per nucleus decreased by 75% in BER inhibited cells 15 min after Fe-ion irradiation. In-track, the mean number of OGG1 foci per micrometre track length was reduced by 25% in the presence of BER inhibitors. As the number of ions traversing each nucleus varies and the length of the tracks per nucleus shows high dispersion (4–20 μm) due to the nuclei’s variable size and shape, we chose not to compare in-track foci per nucleus (Supplementary Table S2), but rather to compare them per track length (Fig. 1C, D, F, and Tables 2 and 3). This makes it easier to perceive differences and is common in the research of DNA damage within heavy-ion tracks^15,32,33^. The number of γH2AX foci per cell also decreased upon BER inhibition, indicating the induction of DSBs by radiation-induced BER processes (Fig. 1D; a summary of the averaged data is shown in Table 2). However, the number of γH2AX foci did not drop to the same extent as OGG1 foci, with a reduction of 13% in-track (foci per tracklength [µm]) and 62% off-track (foci/nucleus). These data were corroborated by live-cell observations of human osteosarcoma cells (U2OS) that express an EGFP-tagged version of the early DSB marker NBS1. In these cells, BER inhibition caused about 26% less radiation-induced NBS1-EGFP foci in Fe-ion tracks and 59% less off-track within 44 min after irradiation (Fig. 1E - F; a summary of the averaged data is shown in Table 3; Supplementary Figure S2 and Supplementary Movie S1).

**Table 2:** OGG1 and γH2AX foci 15 min after Fe-ion irradiation of human fibroblasts (averaged data of Fig. 1C and D).

RIF	RIF location	Control	+ BERi
OGG1	in-track [foci/µm]	1.01 ± 0.05	0.76 ± 0.05
	off-track [foci/nucleus]	12.5 ± 2.9	3.2 ± 1.4
γH2AX	in-track [foci/µm]	0.85 ± 0.03	0.74 ± 0.02
	off-track [foci/nucleus]	5.7 ± 0.7	2.2 ± 0.3

**Table 3:** NBS1 RIF within 44 min after Fe-ion irradiation of human osteosarcoma cells (averaged data of Fig. 1C and D).

RIF location	Control	+ BERi
in-track [foci/µm]	1.52 ± 0.10	1.13 ± 0.06
off-track [foci/nucleus]	14.7 ± 3.1	6.1 ± 1.7

In summary, BER inhibition caused a decrease of OGG1 RIF to a much higher degree off-track than in-track. Concerning DSBs, both γH2AX and NBS1 showed independently that the inhibition of BER caused a decrease of DSBs. Similar to the BER factor OGG1, their decrease was differently pronounced in-track versus off-track. The observed massive reduction of off-track DSBs by around 60–75% caused by BERi clearly demonstrates that BER processes severely add to the DSB load outside the ion track at δ-electron inflicted DNA damage. The detected decrease is in agreement with the BER-inhibitor induced decline of 43% of NBS1 at X-ray-induced DSBs that was observed earlier^20^ and supports findings that indicate that X-ray and δ-electron-induced DSBs are qualitatively alike^20,25^. As BER inhibition caused a decrease of DSBs of up to 26% in-track, we conclude that BER adds to the DSB clustering within heavy-ion tracks.

### Increased resection frequency at Fe-ion-irradiation induced off-track DSBs

Besides the influence of BER on DSB yield and DNA-damage clustering within Fe-ion trajectories, we further explored the influence of in-track clustered DSBs on processing of δ-electron-induced DSBs outside the trajectories. These lesions are considered to be similar to photon-induced DSBs^20,25,34^. Yet, since cells hit by an Fe ion have to cope simultaneously with DNA-damage of different quality (in-track: complex, off-track: less complex) their response at δ-electron-induced DSBs outside the track may be different from their response at X-ray inflicted DSBs. This is supported by earlier results of experiments performing simultaneous irradiation with X-rays and α-particles^26,27^, suggesting a synergistic impact on the DNA damage response (DDR) for mixed irradiation fields in contrast to both radiation qualities alone.

We have previously shown that with increasing DNA-damage complexity an increasing fraction of DSBs becomes resected and relies on this break-end processing for repair^2^. Therefore, in order to find out whether complex in-track DSBs, which frequently become resected, dictate the repair processes of less complex δ-electron-induced DSBs within the same cells, we quantified resected off-track DSBs within cells hit by one or more Fe ions in comparison to X-ray inflicted DSBs. To detect resected DSBs, we co-IF-stained the DSB marker 53BP1 and the resection marker RPA in irradiated cells, which can bind concomitantly to DSBs^35^. Only RPA-positive cells were considered for the analysis. S-phase cells were excluded from the analysis via detection of EdU incorporation.

**Fig. 2:**
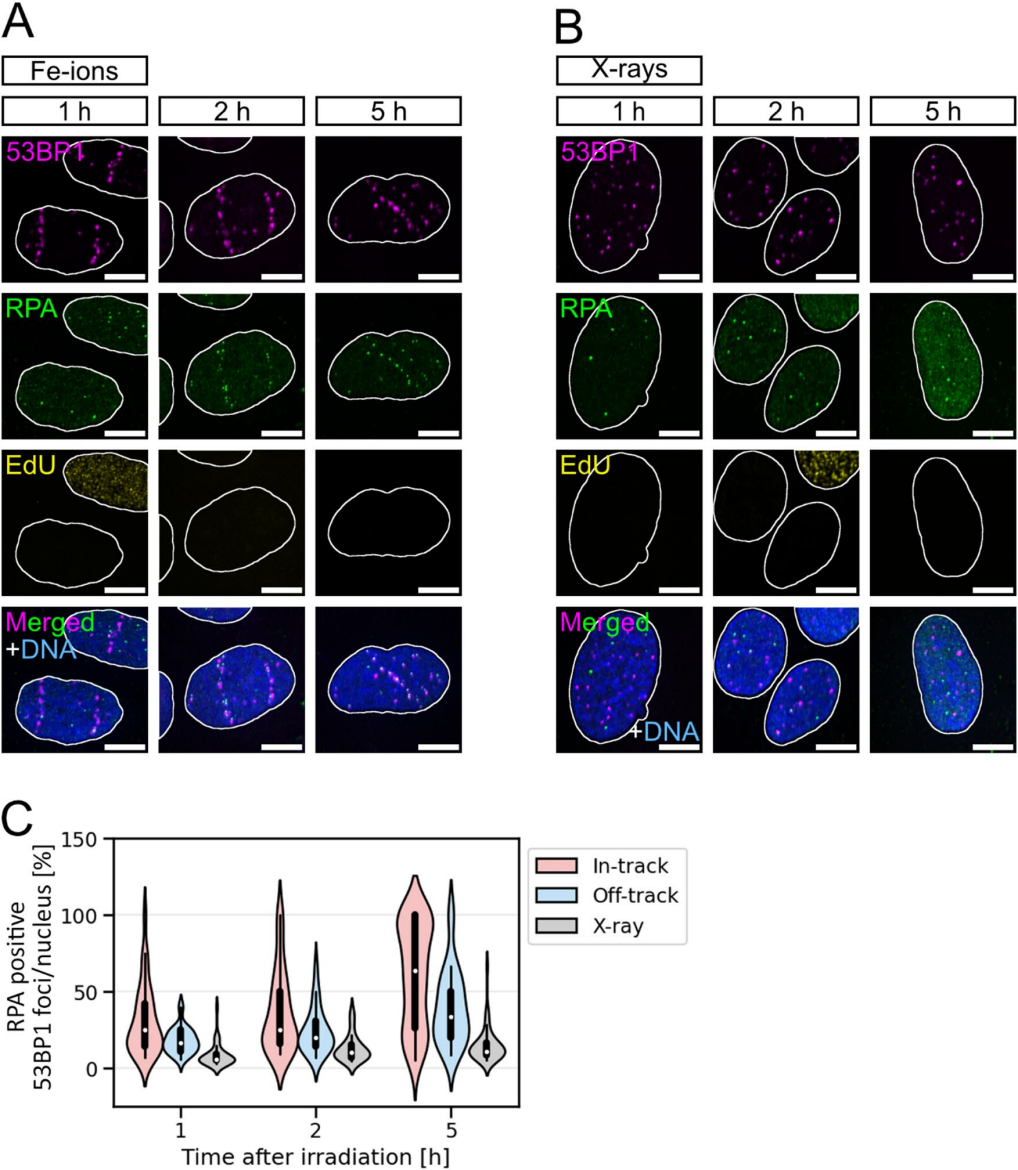
Fe-ion induced off-track DSBs are more frequently resected than those induced by X-rays. (**A & B**) Representative images of osteosarcoma cells (U2OS) that were irradiated with Feions (350 MeV/n, 3 × 10^6^ p./cm^2^) (**A**) or 0.5 Gy X-rays (**B**). To quantify resection-positive DSBs, cells were analysed 1 h, 2 h, and 5 h after irradiation by IF microscopy of RPA (green). DSBs were visualised by 53BP1 IF staining (magenta), S-phase cells via EdU (yellow), and DNA was counterstained with DAPI (blue). Nuclei are indicated by white borders. Shown scale bars indicate 10 μm. (**C**) Distribution of DSB marker 53BP1 foci co-localising with resection marker RPA per respective 53BP1 foci amount in- or off-track (Fe-ion irradiation) or per nucleus (X-ray irradiation). S-phase cells were excluded from the analysis via EdU staining. Only RPA-positive cells were considered for the analysis. Statistical analysis between in- and off-track, and between off-track and X-ray samples was performed by using the nonparametric Mann-Whitney U test: In-track vs. off-track: at 1 h: *p* = 0.0058, 2 h: *p* = 0.065 and 5 h: *p* = 0.00078; Off-track vs. X-ray: at 1 h: *p* = 3.7 × 10^− 8^, 2 h: *p* = 0.00016 and 5 h: *p* = 8.4 × 10^− 10^. With n_Fe ions_=8–46 cells per time point per experiment, N_Fe ions_=2 independent experiments and n_X−rays_=9–32 cell per time point per experiment, N_X−rays_=3 independent experiments.

We found that within a time window of 5 h after irradiation, DSBs within Fe-ion tracks are frequently processed by DNA end-resection (Fig. 2A and C; Table 4). 1 h and 2 h after irradiation approximately one third of in-track 53BP1 foci per cell co-localise with RPA foci. 5 h after irradiation, two-thirds of all in-track 53BP1 foci per cell are decorated with RPA. At the same timepoints, around 20% of off-track 53BP1 foci per cell co-localise with RPA 1 h and 2 h after irradiation and 40% 5 h after irradiation. At all observed timepoints, in-track DSBs seem to be more often resected than off-track DSBs: 73% (1 h) and about 53% (2 h and 5 h) more often. The different frequency of resected DSBs in-track versus off-track reflects their different complexity. Considering the relatively large 53BP1 signal^36^, the high density of DSBs in ion tracks, and the formation of repair centres facilitated by the high damage density^15^, the fraction of resected DSBs in-track may however be overestimated.

At the same time intervals after X-ray irradiation with a dose of 0.5 Gy, the fraction of resection positive DSBs is significantly lower than for off-track DSBs (Fig. 2B and C). At all analysed timepoints after X-ray irradiation, the fraction of resected DSBs (RPA positive) per nucleus is roughly halved compared to off-track DSBs. Since the complexity of δ-electron-induced off-track DSBs and X-ray-induced DSBs is considered comparable^20,25,34^, the observed difference in break-end processing suggests that in cells that were hit by Fe ions the processing of off-track DSBs is affected by the high load of complex DNA-damage within the trajectory.

**Table 4:** Distribution of RPA-positive 53BP1 foci 1 h, 2 h, and 5 h after Fe-ion or X-ray irradiation of human osteosarcoma cells. Only RPA-positive cells were considered. The numbers represent averaged data of Fig. 2C.

Radiation type	RIF location	1 h	2 h	5 h
Fe-ion	in-track/nucleus [%]	31.3 ± 2.4	37.2 ± 3.2	61.2 ± 3.2
	off-track/nucleus [%]	18.1 ± 1.5	24.4 ± 3.0	40.0 ± 3.8
X-ray	nucleus [%]	8.4 ± 1.1	13.2 ± 1.5	15.0 ± 1.5

**Fig. 3:**
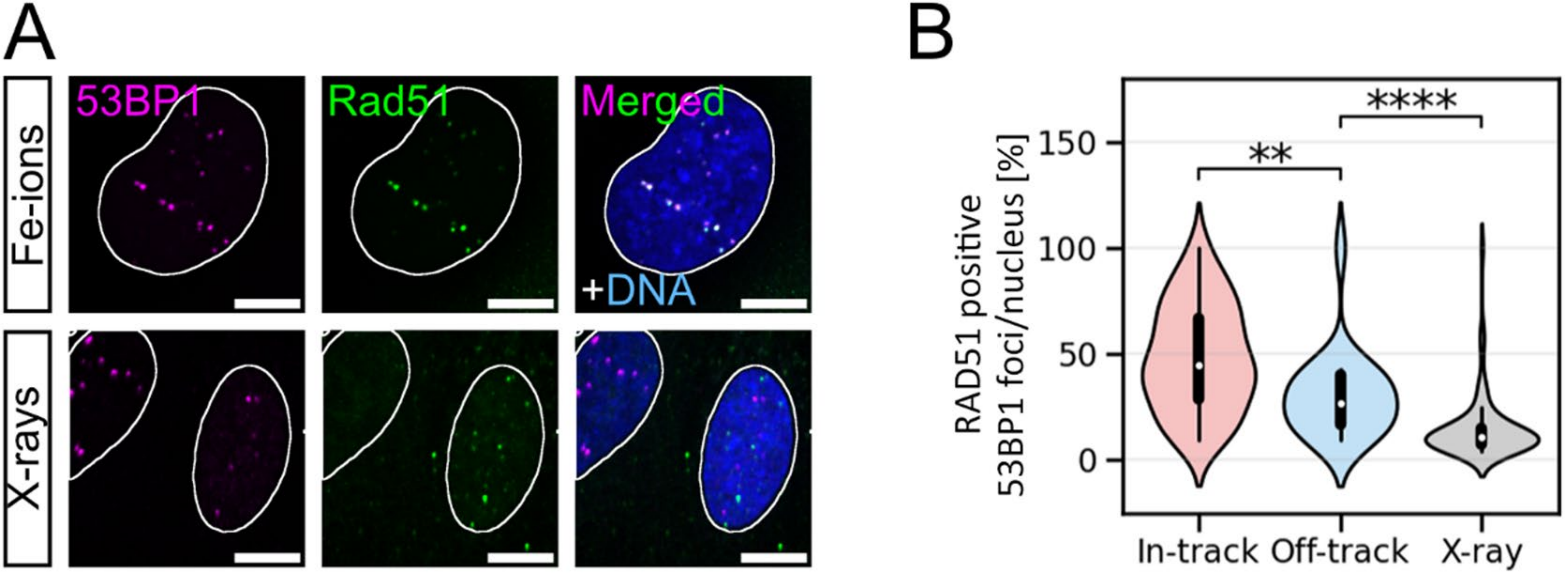
Fe-ion induced off-track DSBs are more frequently repaired by HR than those induced by X-rays. (**A**) Representative images of osteosarcoma cells (U2OS) that were irradiated with Fe ions (350 MeV/n, 3 × 10^6^ p./cm^2^) or 0.5 Gy X-rays. To quantify HR-positive DSBs, cells were analysed 2 h after irradiation by IF microscopy of RAD51 (green). DSBs were visualised by 53BP1 IF staining (magenta), and DNA was counterstained with DAPI (blue). Nuclei are indicated by white borders. Shown scale bars indicate 10 μm. (**B**) Distribution of DSB marker 53BP1 foci co-localising with HR marker RAD51 per respective 53BP1 foci amount in- or off-track (Fe-ion irradiation) or per nucleus (X-ray irradiation). Only RAD51-positive cells were considered. Statistical analysis between in- and off-track, as well as between off-track and X-ray samples was performed by using the nonparametric Mann-Whitney U test: In-track vs. off-track at 2 h: *p* = 0.0019 and off-track vs. X-ray at 2 h: *p* = 1.9 × 10^−6^. With n_Fe ions_=51 cells, N_Fe ions_=1 experiment and n_X-rays_=38–50 cells per experiment, N_X-rays_=2 independent experiments.

HR, which relies on resected ends for the repair-template invasion^6^, is more often used at hight-LET-radiation induced DNA damage^3,4,37,38^. Therefore, we next studied whether the increased frequency of resection indicated by RPA at off-track DSBs compared to X-ray-induced DSBs leads to an increased frequency of DSB repair by HR. Fe-ion or X-ray irradiated cells (U2OS) were IF stained for the HR factor RAD51 and co-IF-stained for the DSB marker 53BP1 (Fig. 3A). Based on the resolution of our microscope and the chosen irradiation dose, these markers can co-localise at DSBs^39,40^. For analysis, the RAD51 decorated 53BP1 foci per RAD51-positive cell were quantified (Fig. 3B; Table 5). Almost half of all in-track 53BP1 foci per cell are RAD51 decorated 2 h post irradiation. Off-track, about one third of the 53BP1 foci per cell is RAD51 positive. The higher fraction of RAD51-positive 53BP1 foci within Fe-ion trajectories is in accordance with the higher DNA-damage complexity and hence increased usage of HR at complex DSBs^3,37,38^. However, similar to the resection data above, the fraction of RAD51-positive DSBs in-track is also affected by the size of the 53BP1 signal, the damage density, and the formation of DNA-repair centres. Interestingly, in X-ray irradiated cells there are 15% RAD51-positive DSBs per cell and thus, about half as many as for off-track DSBs. This implies that in S- and G2-phase cells that were hit by Fe ions, the choice of how to repair off-track DSBs is indeed affected by the load of complex DNA-damage within the trajectory; off-track DSBs seem to be repaired by HR more frequently than X-ray-induced DSBs.

**Table 5:** Distribution of RAD51-positive 53BP1 foci 2 h after Fe-ion or X-ray irradiation of human osteosarcoma cells. Only RAD51-positive cells were analysed. The numbers represent averaged data of Fig. 3B.

Radiation type	RIF location	2 h
Fe ion	in-track/nucleus [%]	48.4 ± 3.0
	off-track/nucleus [%]	30.1 ± 4.3
X-ray	nucleus [%]	14.5 ± 1.5

### Fe-ion induced off-track DSBs are repaired faster than X-ray-induced DSBs in G1-phase cells

Earlier data revealed that in G1-phase cells high-LET-radiation induced DSBs unlike X-ray-induced DSBs are frequently repaired in a resection-dependent manner^2^. This observation and our finding that off-track DSBs in nuclei traversed by swift heavy ions are resected more often than X-ray-induced DSBs (Fig. 2C) suggests that repair of off-track DSBs may also be affected by increased resection frequency in G1-phase cells. We assessed repair kinetics of confluent fibroblasts, mainly G1-cells, and found surprisingly a faster resolving of off-track DSBs as compared to DSB-repair kinetics after X-ray irradiation.

**Fig. 4:**
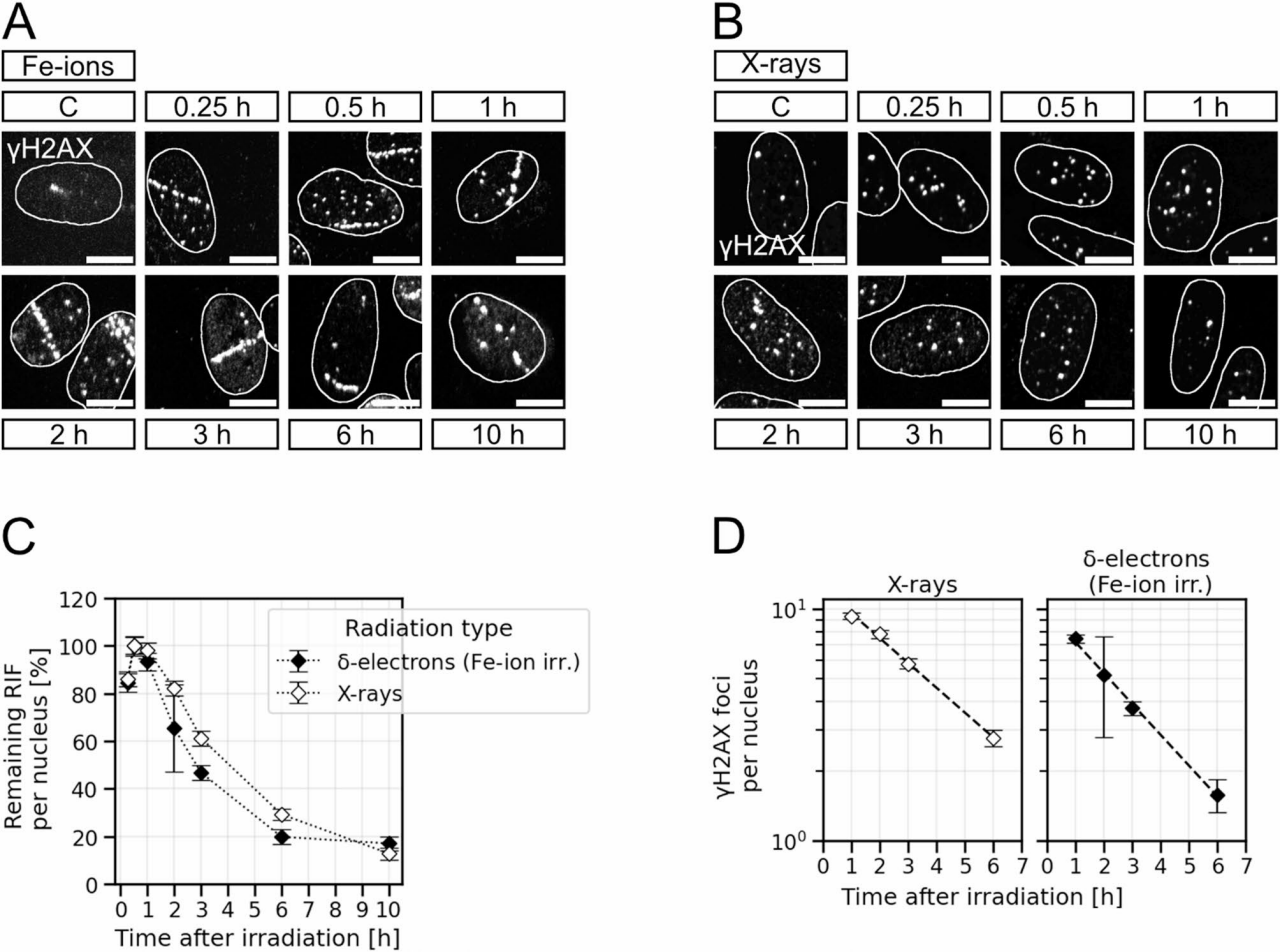
δ-electron generated off-track DSBs within nuclei hit by Fe ions are repaired faster than X-ray induced DSBs. Human fibroblasts were irradiated with Fe ions (350 MeV/n, 3 × 10^6^ p./cm^2^) (**A**) or 0.5 Gy X-rays (**B**). DSB-repair kinetics were generated by quantifying γH2AX foci 15 min, 30 min, 1 h, 2 h, 3 h, 6 h, and 10 h after irradiation. In Fe-ion irradiated cells only off-track foci were quantified, in X-ray irradiated cells all nuclear γH2AX signals were counted. Shown scale bars indicate 10 μm. (**A**) Representative images of cells irradiated with Fe ions. Non-irradiated control cells are indicated by “C”. (**B**) Representative images of cells irradiated with X-rays. Non-irradiated control cells are indicated by “C”. (**C**) The γH2AX assays show the mean percentage of remaining γH2AX foci per nucleus. The numbers of γH2AX foci 30 min after irradiation were considered as the total number of DSBs induced. Data of all other timepoints were normalised by this number. 0 Gy background foci were subtracted. Error-bars indicate the propagation of the standard error of the mean (SEM). (**D**) The logarithm of the mean values of all counts between 1 h and 6 h was fitted via linear regression to obtain and compare the decline: 0.246 h-1 ± 0.009 h-1 and 0.306 h-1 ± 0.012 h-1 for DSB repair after X-ray and Fe-ion irradiation (solely the δ-electron damage), respectively. With n_Fe ions_=41–119 cells per time point per experiment, N_Fe ions_=2 independent experiments and n_X-rays_=50–59 cells per time point per experiment, N_X-rays_=3 independent experiments.

After an initial rise to a maximum number of γH2AX foci 30 min to 1 h after irradiation for both radiation types, the number of δ-electron-induced γH2AX foci per nucleus declines faster than that of X-ray-induced γH2AX foci (Fig. 4A-C). To make sure that the observed difference in repair kinetics is not introduced by the data normalisation, the non-normalised mean values in the time interval from 1 h to 6 h were plotted half-logarithmic (Fig. 4D). This allowed us to fit and compare the difference in slope for both radiation qualities. Similarly to the initial observation, the repair curve of X-ray inflicted DSBs is less steep (slope: −0.246 ± 0.009 h^− 1^) than the repair curve of δ-electron-induced DSBs (slope: −0.306 ± 0.012 h^− 1^), which again indicates that δ-electron-induced DSBs are repaired somewhat faster than X-ray-induced DSBs. Based on the fitted data in Fig. 4D, we calculated the half-time, which determines the time taken for half of the RIF to disappear. It is 2.81 ± 0.11 h for the X-ray and 2.26 ± 0.09 h for the δ-electron data set, respectively, and thus about half an hour less than the time point observed after X-ray irradiation. The 95% confidence intervals for the two fitting parameters do not overlap, suggesting that the difference is significant (Supplementary Figure S2). This indicates that also in G1-phase cells that do not use HR, Fe-ion induced off-track DSBs are processed differently to X-ray-induced DSBs despite their similar quality. The in-track damage load seems to induce a faster or more efficient DDR at off-track DSBs within the same nucleus, which may be based on nucleus-wide activation of ATM and DNA-PK that can be observed with increasing local DNA-damage load^41^. ATM and DNA-PK are central players in activating the DDR^42^ and therefore, a strong activation of these factors may accelerate the DDR at off-track DSBs.

## Discussion

The scope of this work was to investigate the impact of heavy ions on DSB clustering and processing in more detail. On the one hand, we focused on how base damage induced by heavy ions influenced the overall DSB load. On the other hand, we focused on the mixed radiation quality of high energetic heavy ions to observe the impact of the intrinsic mixed radiation quality of Fe-ion irradiation on the repair of δ-electron inflicted DSBs.

To study the impact of BER on the DSB load after heavy-ion irradiation, human fibroblasts were pre-incubated with two inhibitors, methoxyamine and TH5487, and compared to non-treated control cells regarding the distribution of the BER glycosylase OGG1, as well as the amount of DSBs assessed by co-staining with γH2AX. The effect of the BER-inhibitor combination was assessed by treating cells with the strongly oxidising agent KBrO_3_, which showed a well pronounced effect on the reduction of OGG1 and γH2AX during inhibition (Table 1, Supplementary Figure S2), supporting its usability for inhibiting the BER pathway for the irradiation experiments.

The inhibition of the BER pathway led to significantly decreased OGG1, γH2AX, and NBS1 RIF in the trajectory and the off-track region around the track of swift Fe ions (Tables 2 and 3). This indicates that the processing of in- and off-track Fe-ions-originated base damage causes additional DSBs leading to increased DSB clustering, which is of relevance for the biological effect of HZE particles in space radiation. This finding together with the one of Yang et al. on X-ray induced base damage^19^ could serve as input for radiobiological modelling of DNA-damage induction and repair^13,43–45^.

Comparing the DSB markers γH2AX and NBS1 (Tables 2 and 3), the decrease of in-track γH2AX foci upon BER inhibition was half of the one extracted from the NBS1-data, i.e. about 13% for γH2AX and 26% for NBS1, respectively. At off-track damage, however, the decrease was quite similar, about 39% for γH2AX and about 42% for NBS1 (Tables 2 and 3). This observation is based on the fact that NBS1 foci are smaller than those of γH2AX^20^. NBS1’s smaller (and hence less overlapping) dimensions offer a more precise distinction of single foci especially in the lesion-dense ion trajectory and thus allow an improved observation of changes in the foci and DSB numbers due to BER inhibition. Interestingly, the observed impact of BER inhibition on the DSB number visualised by NBS1-GFP recruitment in the trajectory is not reflected in an influence on the recruitment kinetics of the repair factor NBS1 observed earlier^20^. The lack of influence on the recruitment kinetics was suggested to be caused by the dominance of prompt DSBs, i.e. directly induced DSBs that are induced independently of BER actions^20^. Our finding that BER inhibition caused a drop of in-track DSBs by only 26% versus 42% off-track supports this assumption.

The difference in the observed ratio of OGG1 foci and additional BER induced DSBs in- and off-track (Tables 2 and 3) most likely is based upon the high damage density and thus strong damage clustering in the track region. It was shown earlier that due to the high clustering of DNA damage in ion tracks, repair of these lesions is slowed down, including base lesions visualised by OGG1^28^. Thus, as their repair is hampered, inhibiting repair of base damage would have fewer consequences on the number of extra DSBs. Furthermore, clustering and the possible formation of repair centres of in-track RIF impedes to differentiate whether one focus contains several DSBs as suggested for high-LET ion tracks in several studies^13–15,33,46,47^. Taking a closer look at OGG1, the smaller effect of its inhibitor on its binding to in-track DNA damage compared to off-track damage (Table 2) may be further due to increased molecular crowding at in-track DNA damage^48^. Increased crowding because of DNA-repair factors recruited to areas of dense DNA damage may prevent the release of OGG1 from its target, thus causing a smaller effect by the OGG1 inhibitor TH5487. Taking into account the optical resolution limit of the microscope and the mean focus diameter, additional emerging DSBs in the track region may become undetectable within our experimental setup. This accounts especially for γH2AX as the phosphorylation of H2AX extends several mega bases around a DSB^49^. Future studies using an increased resolution can improve the analysis of BER induced DSB formation, especially along the densely ionising ion track. Emerging techniques like expansion microscopy, which rely on the physical isotropic expansion of the specimen, could in principle enlarge the track area while maintaining the spatial antibody context^50,51^. Although, difficulties may occur at the evaluation step due to the subdivided structure of foci of common DSB markers like γH2AX and 53BP1 into nano-foci observed with increased resolution, while the correlation between the amount of nano-foci and DSBs is still unclear^52–54^.

In this study, a special focus was put on the spatial distribution of DSB repair after Fe-ion irradiation as a representative of the HZE particle encounter of GCR. We found an increased frequency of resection-use at δ-electron-induced DSBs in nuclei traversed by Fe ions in comparison to X-ray-induced DSBs (Table 4). As DSBs induced by δ-electrons are assumed to match in their complexity with photon-induced DSBs^20,25,34^, the finding suggests that the damage quality of each single off-track DSB within an Fe-ion hit nucleus is no crucial factor in the decisions of its repair processing. In contrast, it seems that in the presence of a high load of clustered in-track DNA damage the entire DDR within a cell is shifted towards repair pathways that require resection. The possibility of influencing the DSB-repair choice in *trans* was observed earlier. In γ-irradiated mammalian cells linearised plasmid-DNA transfected into the cells after irradiation was far more often repaired by microhomology-mediated aEJ than in non-irradiated cells^55^. Our findings on the usage of resection-dependent HR support this notion even further. In comparison to X-ray irradiation, the observed fraction of RAD51 decorated DSBs is not only far higher in-track where DNA damage is of high complexity and the observation is in line with earlier data^3,37^, but notably also off-track where damage complexity equals the one of X-ray-induced lesions (Table 5). While RAD51 dependent HR is used in S and G2 phase only, we know from earlier data that heavy-ion induced DSBs also rely on resection-dependent repair in G1 phase^2^. Therefore, one can assume that also in G1-phase cells in-track DNA damage dictates resection and repair processes at off-track DSBs with severe consequences, as resection-dependent repair, like microhomology mediated aEJ in G1 phase is considered error-prone^56^.

Since the DNA damage after heavy-ion irradiation is composed of highly complex in-track damage with many lesion clusters and less complex off-track damage, it depicts the situation of a simultaneous mixed beam irradiation. Studies observing the DSB repair after synchronised X-ray and α-particle irradiation suggest a non-additive effect on the number of RIF as well as on repair kinetics of those lesions in contrast to each single radiation quality alone^26,27^. Indeed, in cells hit by Fe ions we found the repair kinetics of δ-electron-induced DSBs did not match the one of X-ray-induced DSBs (Fig. 4). However, while Sollazzo and co-authors described a slower progression in repair kinetics of simple DNA breaks upon concomitant alpha- and X-ray irradiation in comparison to X-ray irradiation only^26^, we observed a slightly faster repair of off-track DSBs in comparison to X-ray-induced DSBs. Our findings are in line with Nakajima and co-authors^25^, whose data on Fe-ion or X-ray irradiated confluent G0/G1-phase fibroblasts also indicate a faster repair of DSBs induced by δ-electrons^25^. Despite the divergencies, all these data show that the repair processes of less complex DSBs are affected by DSBs of high complexity within the same cell.

Based on the observation that resection-related factors such as RPA and RAD51 are recruited to δ-electron-induced DSBs, which are associated with slower DSB-repair mechanisms such as HR or aEJ, it is unlikely that the observed faster repair kinetics of δ-electron-induced DSBs are based on the choice of DSB-repair pathway. One reason for the faster repair kinetics of this damage compared to X-ray-induced DNA lesions may be the faster or more efficient initiation of the DDR in cells traversed by heavy ions. Due to their DNA-damage load, ATM and DNA-PK are activated throughout the nucleus of these cells^41^, which may cause a robust and accelerated induction of DNA-repair processes.

Taken together, our results emphasise the importance of studying the additional δ-electron inflicted DNA damage after heavy-ion encounters. Not only can the δ-electron range reach up to the mm-scale^34^ and thus add to the dose distribution in adjacent cells not initially hit by the ion, BER processes seem to further increase the total amount of DSBs through the concurrent processing of irradiation induced base lesions along the track as well as off-track and thus in cells exposed to long range δ-electrons. Importantly, the simultaneous occurrence of a high complex damage load in cells directly traversed by heavy ions seem to impact the overall DDR in these cells. Foremost the increase of resection at not only high complex lesions (in-track) but also at simpler DSBs around the track region (off-track), which resemble photon-induced DSBs can be of interest regarding the higher frequency of chromosomal modifications after heavy-ion irradiation. Taken together, interaction of DNA-damage processing is relevant for DNA repair and consequently cancer development.

## Materials and methods

### Cell culture and inhibitor treatment

Primary human foreskin fibroblasts (AG1522D, Coriell Institute), human osteosarcoma cells (U2OS, ATCC (American Type Culture Collection)), and U2OS-NBS1-2GFP, kindly provided by Claudia Lukas (The Novo Nordisk Foundation Center for Protein Research, University of Copenhagen, Copenhagen, Denmark)^57^, were kept at 95% humidity, 5% CO_2_ and 37 °C in Dulbecco’s Modified Eagle Medium (DMEM) with 4.5 g/l glucose, 15% fetal bovine serum (FBS) (AG1522D) or 10% FBS (U2OS, U2OS-NBS1-2GFP), 1% non-essential amino acids (AG1522D), and 400 µg/ml Geneticin (G418) and 1 µg/ml Puromycin (U2OS-NBS1-2GFP cells only). AG1522D fibroblasts were accumulated in G1 phase via contact inhibition (CPD in the range of 20–30).

For experiments, cells were seeded either in single 35 mm Petri dishes and/or on #1.5 24 × 24 or 18 × 18 mm glass cover slips for the fixed cell experiments or in chambered coverslips (#1.5) with four wells (ibidi) for live cell imaging.

To block the BER pathway, a combination of two inhibitors was used in the cells-culture medium: 10 µM TH5487, an OGG1 inhibitor that prevents OGG1’s binding and repair of 8-oxoG^58,59^ and 20 mM methoxyamine (MX), an indirect APE1 inhibitor, as it makes apurinic and apyrimdinic sites insensitive to APE1^60^. Control cells were treated with DMSO. Incubation time was at least 1 h at 37 °C prior irradiation and the inhibitor mixture was prepared shortly before performing an experiment.

In order to test the chosen BER inhibitors and the ability of BER to induce DSBs, we generated DNA-base damage by incubating the cells for 1 h with 40 mM KBrO_3_ in culture medium under cell-culture condition (95% humidity, 5% CO_2_ and 37 °C).

### Cell irradiation

X-ray irradiation of AG1522D fibroblasts and U2OS cells was performed with a dose of 0.5 Gy and a dose-rate of approximately 2.5 Gy/min. The X-ray tube’s peak voltage (Isovolt Titan; GE Sensing and Inspection Technologies) was set at 250 kV along with the tube current at 16 mA. Filtering was done by 1 mm Copper and 1 mm Aluminium. The resulting LET is around 2 keV/µm.

Iron (Fe) ion irradiation was performed at the GSI synchrotron SIS18 at an energy of 350 MeV/n and a fluence of 3 × 10^6^ particles/cm^2^. The resulting LET was around 225 keV/µm yielding a dose of 1 Gy. To visualise DNA damage along the ion traversal, cell cultures were irradiated horizontally with a low angle (2°). Non-irradiated control samples have been carried to the cave as well.

### Live cell imaging

Live cell imaging of U2OS-NBS1-2GFP in glass-chamber slides was performed after Fe-ion irradiation. To prevent the dose contribution of adjacent chambers, only the outer chambers have been irradiated and imaged, while imaging was initiated 1 min before irradiation and continued for 44 min afterwards. Per well, 10–15 different locations were imaged sequentially with the frequency of 1 Z-stack/min per location.

Images of living cells at the beam line were acquired using a modified remote-controlled OLYMPUS IX73 microscope equipped with an UPlanFL60x/1.2 water or 100 ×/1.4 oil immersion lens and a 1.6 x optovar, an Andor Ixon Ultra DU-888 EM-CCD camera and Andor iQ 3 version 3.6.1 (Andor Technology).

### Immunofluorescence microscopy

For immunofluorescence microscopy, cells were fixed in 2% formaldehyde in phosphate buffered saline (PBS) (prepared from paraformaldehyde, AppliChem, Darmstadt, Germany) for 15 min and subsequently permeabilised in 0.5% Triton-X 100 (Carl Roth, Karlsruhe, Germany) in PBS for 10 min. Before antibody staining, the fixed samples were blocked with 0.4% bovine serum albumin (BSA; Carl Roth, Karlsruhe, Germany) in PBS for 20 min at room temperature. After blocking, cells were stained with different primary antibodies diluted in 1x PBS, 0.4% BSA: anti-γH2AX (1:500, Millipore, clone JBW301, 1 mg/ml), anti-CENPF (1:750, Novus Biologicals, 1 mg/ml), anti-RPA (32 kDa subunit, 1:300, Santa Cruz, clone 9H8, 200 µg/ml), anti-53BP1 (1:500, Calbiochem), anti-RAD51 (1:200, Abcam, clone 14B4, 1 mg/ml) and anti-OGG1 (1:500, Novus Biologicals, 1 mg/ml). Alexa fluor antibodies from goat against rabbit or mouse were used as secondary antibodies (1:400, Invitrogen Alexa Fluor™ 488 and 561 (2 mg/ml)). DNA was counterstained with 4,6-Diamidin-2-phenylindol (DAPI; AppliChem, Darmstadt, Germany) and mounted with SlowFade™ Diamond Antifade Mountant (Invitrogen).

EdU Click-647 ROTI^®^kit for Imaging (Carl Roth, Karlsruhe, Germany) was used according to the manufacturer to exclude S-phase nuclei.

Images were acquired using a Nikon Eclipse Ti spinning disk confocal microscope equipped with a 100x objective (oil immersion) and Andor iQ 3 version 3.6.1 (Andor Technology) or a Leica TCS SPE confocal microscope equipped with a 63x objective (oil immersion) together with the Leica Application Suite X (LAS X) imaging software (Leica Microsystems).

### Statistics and reproducibility

In all violin plots, the width of the density estimation represents the probability of all values within the data set. The box inside the kernel density estimation represents the interquartile range with the median depicted as white dot; the whiskers cover the 1.5x interquartile range. Statistical analysis was performed by using the nonparametric Mann-Whitney U test with the significance level of 0.05. Sample size is indicated by the number of cells (n) and the number of independent experiments (N), which were performed on different days and with a different batch of cells obtained from the same origin. Unless otherwise indicated, error bars indicate the standard error of the mean of single-cell data.

## Supplementary Information

Below is the link to the electronic supplementary material.

Supplementary Material 1

Supplementary Material 2

## Data Availability

Datasets that led to the conclusions within this study are available from the corresponding author upon reasonable request.
