# Correction: Base-excision repair increases DNA double-strand break clustering within heavy-ion tracks and modulates repair at δ-electron-induced breaks

**DOI:** 10.1038/s41598-026-39156-5

**Published:** 2026-02-16

**Authors:** Laura Schwan, Nicole B. Averbeck, Marco Durante, Burkhard Jakob

**Affiliations:** 1 Department of Biophysics, GSI Helmholtzzentrum für Schwerionenforschung GmbH, Planckstr. 1, 64291 Darmstadt, Germany; 2 Department of Biology, Technische Universität Darmstadt, Darmstadt, Germany; 3 Department of Condensed Matter Physics, Technische Universität Darmstadt, Darmstadt, Germany

Correction to: *Scientific Reports* 10.1038/s41598-025-32823-z, published online 09 January 2026

The original version of this Article contained errors.

In the Materials and methods section, subsection Cell culture and inhibitor treatment the sentence:

“To block the BER pathway, a combination of two inhibitors was used in the cells-culture medium: 10 µM TH5487, an OGG1 inhibitor that prevents OGG1’s binding and repair of 8-oxoG^58,59^ and 40 mM methoxyamine (MX), an indirect APE1 inhibitor, as it makes apurinic and apyrimdinic sites insensitive to APE1^60^.”

now reads:

“To block the BER pathway, a combination of two inhibitors was used in the cells-culture medium: 10 µM TH5487, an OGG1 inhibitor that prevents OGG1’s binding and repair of 8-oxoG^58,59^ and 20 mM methoxyamine (MX), an indirect APE1 inhibitor, as it makes apurinic and apyrimidinic sites insensitive to APE1^60^.”

The original Article has been corrected.

