# Supplementary material for: Base-excision repair increases DNA double-strand break clustering within heavy-ion tracks and modulates repair at δ-electron-induced breaks
Source: Sci Rep. 2026 Jan 9;16:1339. doi: 10.1038/s41598-025-32823-z (PMC12796214; doi:10.1038/s41598-025-32823-z)
Supplement: Supplementary file 2 — Supplementary Material 2 [file 41598_2025_32823_MOESM2_ESM.pdf]

Supplementary Information

Base-excision repair increases DNA double-strand break clustering within heavy-ion tracks and modulates repair at δ-electron-induced breaks

Laura Schwan[1,2,4], Nicole B. Averbeck[1,4,*], Marco Durante[1,3], Burkhard Jakob[1,2]

[1]Department of Biophysics, GSI Helmholtzzentrum für Schwerionenforschung GmbH, Darmstadt, Germany

[2]Department of Biology, Technische Universität Darmstadt, Darmstadt, Germany

[3]Department of Condensed Matter Physics, Technische Universität Darmstadt, Darmstadt, Germany

[4]These authors contributed equally to this work.

[*]Corresponding author
Contact information:

n.averbeck@gsi.de
GSI Helmholtzzentrum für Schwerionenforschung GmbH
Department of Biophysics
Planckstr. 1
64291 Darmstadt
Germany

Supplementary Figure S1

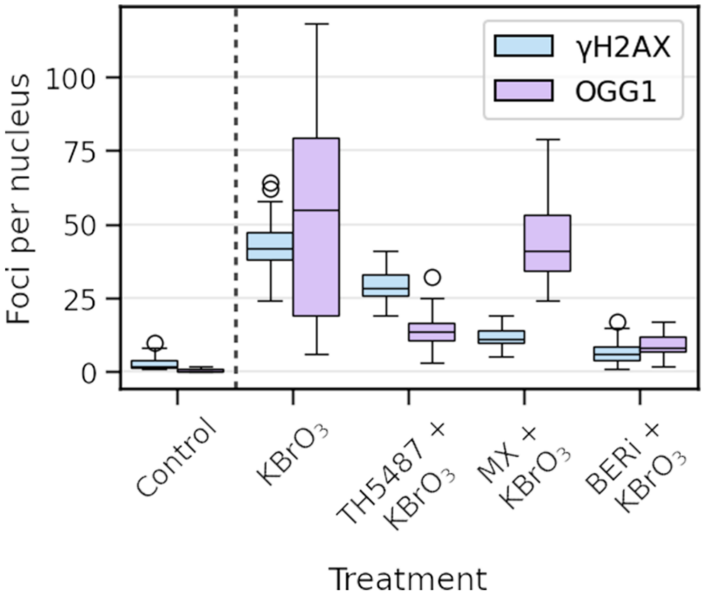

Figure S1: Number of γH2AX and OGG1 foci in normal human fibroblasts without inhibitor ($KBrO_3$), with OGG1 inhibitor TH5487 (TH5487 + $KBrO_3$), with APE1 inhibitor methoxyamine (MX + $KBrO_3$), or with the combination of both (BERi + $KBrO_3$) 1 h after $KBrO_3$ incubation. Control samples without inhibitor and without $KBrO_3$ treatment are on the left to the dashed line. The box ranges from the first to third quartile while the whiskers extend to the 1.5x inter quartile range (ICR), with outliers outside of this range represented as circles and the line within the box as median. With n=25-30 cells per treatment per experiment, N=1-2 independent experiments.

Supplementary Figure S2

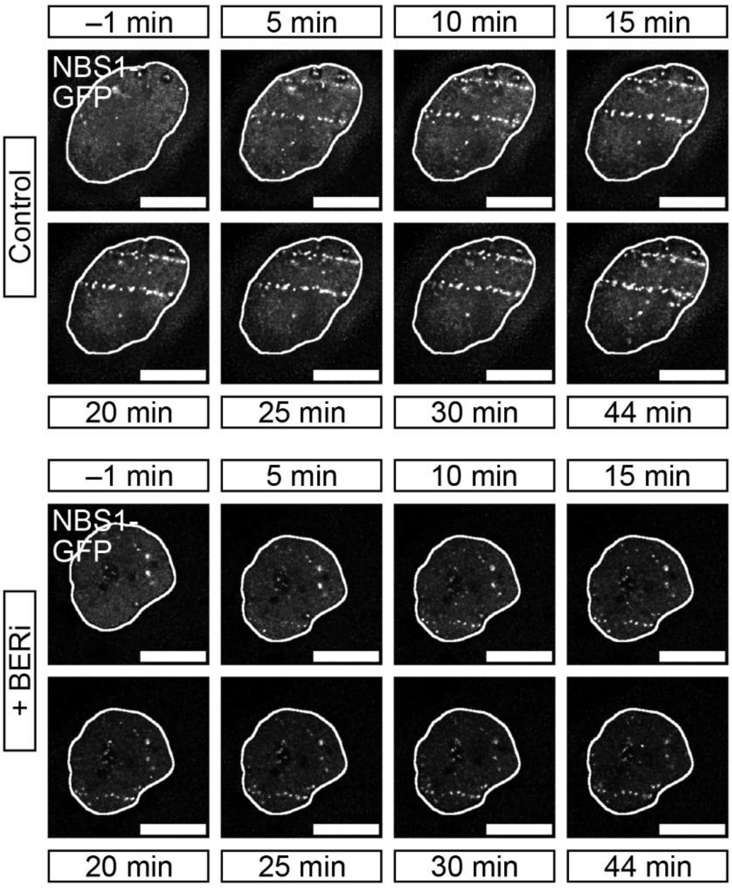

Figure S2: Human osteosarcoma cells expressing GFP-tagged NBS1 were treated with (+ BERi) or without (control) BER inhibitors and irradiated with Fe-ions (350 MeV/n, $3x10^6$ p./cm^2). The cells were observed from 1 min before until 44 min after irradiation. Shown are images before (-1 min) and at different times after irradiation. The entire movie is deposited in Supplementary Movie S1. The scale bars indicate 10 µm.

Supplementary Figure S3

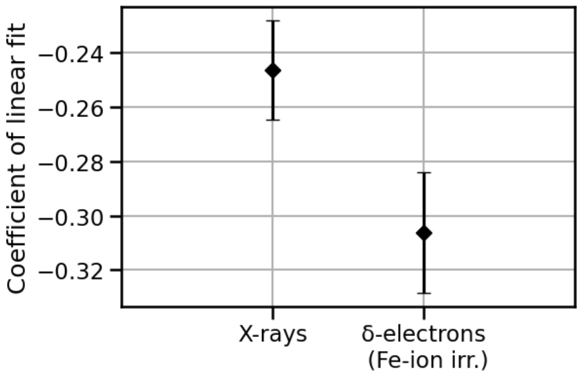

Figure S3: The first coefficient (a) of the linear regression (ax+b) of the logarithmically transformed mean γH2AX foci count/nucleus between 1 h and 6 h after X-ray or Fe-ion irradiation (only δ-electron induced off-track DSBs). Error bars are within the 95% confidence interval.

Supplementary Table S1

Supplementary Table S1: Fe-ion-irradiation induced in-track foci per nucleus of OGG1, γH2AX and NBS1. To study the occurrence of foci in relation to functional base excision repair (BER), the cells were treated with (BERi) or without BER inhibitors (control). OGG1 and γH2AX foci were analysed 15 min after irradiation of human fibroblasts (data are based on results shown in Figure 1C and D) and NBS1 foci were quantified within 44 min after irradiation of living human osteosarcoma cells that express GFP-tagged NBS1 (data are based on results shown in Figure 1F).

RIF	Control [in-track foci/nucleus]	+ BERi [in-track foci/nucleus]
**OGG1**	10.8 ± 0.5	8.7 ± 0.6
**γH2AX**	9.1 ± 0.5	8.3 ± 0.4
**NBS1**	36.6 ± 4.5	30.1 ± 4.8
